# Cost Effectiveness and Impact in Quality of Care of a Pediatric Multidisciplinary Stone Clinic

**DOI:** 10.1097/pq9.0000000000000474

**Published:** 2021-09-24

**Authors:** Jessica M. Ming, Roberto I. Lopes, Elizabeth A. Harvey, Michael E. Chua, Megan A. Saunders, Mina Matsuda-Abedini, Darius J. Bägli, Walid A. Farhat, Joana Dos Santos

**Affiliations:** From the * Division of Urology, The Hospital for Sick Children and Department of Surgery, University of Toronto; Toronto, ON, Canada; † Division of Nephrology, The Hospital for Sick Children and Department of Pediatrics, University of Toronto, Toronto, ON, Canada.

## Abstract

Herein, we assess the cost-effectiveness of a multidisciplinary clinic for children with urinary stones. The clinic’s primary goals were to decrease unnecessary visits, imaging, and costs while optimizing the quality of care.

**Methods:**

Between October 2012 and January 2016, children with complex stone disease, previously treated in urology and/or nephrology clinics, were seen at a triannual pediatric combined stone clinic. We compared the number and cost of ultrasounds, emergency room (ER) visits, and stone surgeries performed before and after each patient’s initial evaluation. All patients received satisfaction surveys.

**Results:**

Among the 79 patients, 27 were seen at least twice in the combined clinic and followed multiple times in either urology or nephrology clinics. The mean number of ER visits per patient per year significantly decreased from 0.29 ± 0.36 to 0.10 ± 0.15 (*P* = 0.002). The mean cost of ER visits went from CAD$ 23.44 ± 28.80 to CAD$ 4.14 ± 12.18 (*P* = 0.002). Likewise, the mean annual number and cost of stone-related surgeries significantly decreased [(0.38 ± 0.63 versus 0.20 ± 0.32 after the MSC started (*P* = 0.026) and mean annual cost of surgeries went from CAD$ 182.97 ± 301.49 to CAD$ 41.59 ± 110.97 (*P* = 0.022)]. Among the survey responses returned, 75% of families believed the clinic was time-saving.

**Conclusions:**

Despite a small sample size, the number of ER visits and stone-related operations significantly decreased after the initial combined clinic intervention. Longer-term data will hopefully confirm if the positive findings continue.

## Abstract 2

Supplemental Digital Content is available in the text.

## INTRODUCTION

Pediatric nephrolithiasis is a diagnosis increasingly seen in pediatric urology and nephrology clinics. Over the past 25 years, stone disease in children has been rising by approximately 6%–10% each year.^1,2^ Among those with stone disease, at least 70% of children will have an associated metabolic abnormality identified in their urine.^3,4^ Rates of recurrence of urolithiasis range from 16% to 44%.^5,6^ For patients at risk for chronic high stone burdens, such as cystinuria and primary hyperoxaluria, a multidisciplinary approach becomes of utmost importance to optimize outcomes for these patients. A team consisting of dietitians, social workers, nurses, nurse practitioners, and medical and surgical physicians allows the creation of an inclusive treatment plan intended to enhance medication adherence, dietary changes, and lifelong strategies to prevent further stone disease. The cost of nephrolithiasis to the healthcare system is rising as the incidence of stone disease increases.^7^ In a tertiary care pediatric institution with a single payer healthcare system, this increase in cost must be addressed while working with an already limited budget. There are limited reports addressing the management of these patients using a multidisciplinary approach. Here, we assess the cost-effectiveness of a multidisciplinary clinic for children with urinary stones in the Canadian healthcare system. Before the multidisciplinary stone clinic (MSC), patients with complex stone burden in our institution were seen separately in Urology and Nephrology clinics, often with a short interval between appointments and redundant imaging studies. The MSCs primary goals were to decrease unnecessary visits, imaging, and ultimately cost while optimizing quality of care. In this study, we aim to determine if the MSC clinic goals were achieved.

## METHODS

In October 2012, we initiated an MSC involving the pediatric urology and nephrology staff physicians along with a dietician. The MSC targeted patients with complex stone histories, potentially requiring both surgical intervention and medical management. Complex stone patients are defined by one or more of the following characteristics: recurrent urolithiasis, recurrent stone-related surgical procedures, and metabolic disorders associated with high stone burden (ie, cystinuria, primary hyperoxaluria, Lesch Nyhan syndrome).

The clinic was held every 4 months in a common setting. Following approval by our institutional Quality Improvement (QI) committee, we evaluated all patients seen in the clinic from October 2012 until January 2016. Baseline patient characteristics were collected, including age, gender, diagnosis, etiology of stone disease (when known), and duration in MSC versus independent clinics. Electronic and paper charts were reviewed to track patient progress. We compared the number and cost of imaging studies, stone-related emergency room (ER) visits, and stone surgeries performed at least 2 years before and after the initial evaluation of each patient in the clinic. We used the 2 years before stone clinic as comparative information in the analysis. At the time of data analysis, the MSC was in place for 12 months. To compare with clinics before the MSC, we corrected the follow-up time out to 2 years. We used 12 months as follow-up time and averaged data per year for 2 years. The hospital billing department provided billed charges for all requested services. Renal ultrasounds are billed at CAD$150.60, a physician ER visit CAD$80.00, and cystoscopy with ureteroscopic stone case at CAD$482.40. Comparisons were made using a nonparametric test, the Wilcoxon Signed-rank test, and statistical significance was set at *P* value <0.05. Descriptive statistical analysis was done using Microsoft Excel and SPSS v26 statistical programs.

All patients received satisfaction surveys in the clinic at the time of their visit or via mail (**Figure 1, Supplemental Digital Content 1**, which describes survey of MSC handed out in person or sent to families for evaluation, http://links.lww.com/PQ9/A312). Satisfaction surveys were used to assess compliance and patient perceptions of the quality of care provided in the MSC. Parents or the patients answered questions regarding their adherence to prescribed fluid intake, dietary changes, and medications, if any. They were also asked about MSC clinic flow, consistency of information provided by the specialty teams, and whether they believed the MSC was time-saving for parents and patients.

This QI project was approved by our institutional QI committee.

## RESULTS

Since MSC initiation, 79 patients were evaluated. Among the 79 patients, 32 were seen at least twice in the MSC (followed for at least 6 months). Before the combined clinic, 27 of these patients were followed multiple times in either a urology or nephrology clinic; 5 patients were directly referred. The median duration of follow-up for individual clinics was 36 (IQR 7.5–39) months, whereas for MSC it was 12 (IQR 6–18) months. This subset of children was analyzed to compare the absolute numbers and costs of ultrasounds, ER visits, and urologic surgeries performed before and after the initiation of the MSC. The median age at diagnosis of all clinic patients was 72 months (range 5–185 months). Among all clinic patients, an underlying metabolic disease was identified in 14 (51.9%), including idiopathic hypercalciuria (25.9%), cystinuria (18.6%), and primary hyperoxaluria (7.4%) (Table 1).

**Table 1.** Baseline Characteristics for Our Cohort (n = 27)

Median age (months) 72 (range 5–185) 14 (51.9%)
Underlying metabolic disease (%)
Idiopathic hypercalciuria 7 (25.9%)
Cystinuria 5 (18.5%)
Primary hyperoxaluria 2 (7.4%)
No metabolic diagnosis 7 (25.9%)
Significant risk factor (%) 6 (22.2%)
Juvenile osteoporosis 1 (3.7%)
Juvenile arthritis 1 (3.7%)
Postchemotherapy 1 (3.7%)
Nephrocalcinosis 3 (11.1%)

The mean number of annual ultrasounds performed before MSC was 1.97 *±* 0.76 compared to 1.60 *±* 0.71 after (*P* = 0.066). The mean annual billing costs for ultrasounds was CAD$ 296.01 *±* 115.19 before being seen in MSC compared to CAD$ 241.48 *±* 109.08 after MSC clinic participation (*P* = 0.050). The mean number of ER visits per patient per year showed a statistically significant decrease from 0.29 *±* 0.36 to 0.10 *±* 0.15 (*P* = 0.002). The mean billing costs for these ER visits went from CAD$ 23.44 *±* 28.80 to CAD$ 4.14 *±* 12.18 (*P =* 0.002). Urologic surgeries directly related to stone disease had a statistically significant decrease in the annual number of surgeries and cost. The mean annual number of surgeries per patient was 0.38 *±* 0.63 versus 0.20 *±* 0.32 after the MSC started (*P* = 0.026), and the mean annual cost of surgeries related to stone disease changed from CAD$ 182.97 *±* 301.49 to CAD$ 41.59 *±* 110.97 (*P* = 0.022). After correction for a follow-up time of 2 years, the results remained significant (Table 2).

**Table 2.** Impact of the Stone Clinic Project on Urinary Tract Ultrasound, ER Visits, and Stone Surgery

Number per Year (Cost)	Before Clinic Median (IQR)	After Clinic Median (IQR)	*P* *
Ultrasound	1.97 *+* 0.76	1.60 *+*0.71	0.066
	(296.01 *+* 115.19 CAD)	(241.48 *+* 109.08 CAD)	(0.050)
ER visit	0.29 *+* 0.36	0.10 *+* 0.15	0.002
	(23.44 *+* 28.80 CAD)	(4.14 *+* 12.18 CAD)	(0.002)
Stone surgery	0.38 *+* 0.63	0.20 *+* 0.32	0.026
	(182.97 *+* 301.49 CAD)	(41.59 *+* 110.97 CAD)	(0.022)

Before clinic: Patients followed at Nephrology and/or Urology clinics for at least 2 years before the creation of the MSC. After clinic: Patients followed at the MSC for 12 months. Follow-up time has been corrected for 2 years.

*Analysis using the Wilcoxon Sign Rank test for paired nonparametric distribution.

Twenty-four out of 79 (30.3%) surveys were returned. Seventy-five percent of families believed the clinic was time-saving and 79% agreed that the information provided was consistent between the teams resulting in a better understanding of their child’s condition.

## DISCUSSION

As the prevalence of metabolic stone disease increases in the pediatric population and increasing importance is placed on the efficiency of healthcare costs, we describe a feasible approach to treat pediatric stone disease. By taking a multidisciplinary approach, children with defined metabolic disease receive cohesive and satisfying treatment for their condition. To our knowledge, there are only a few reports describing the outcomes of such a metabolic stone clinic.

In 2005, Nakada et al^8^ described a metabolic stone clinic to treat patients with cystinuria. They identified 20 patients and found that improved adherence to medical management with a combined team approach significantly reduced the number of stone recurrence rates and surgical interventions needed to treat stones. Similarly, we observed a significant reduction in required surgeries after patients had received optimal medical management, and adequate counseling from multiple teams. The present study suggests that patients with other metabolic abnormalities may benefit from such a clinic.

Our findings are similar to those observed by Naqvi et al.^9^ They evaluated a metabolic stone population in children from a developing country and included a much larger population of 2,618 patients. The group identified the most common etiologies of metabolic stone disease in Pakistan. Given that stone disease can go undetected for an extended period of time, they described a system to stratify treatment for children presenting with acute disease. Using a multidisciplinary approach, which included aggressive medical or behavioral interventions, they were able to preserve the renal function of children and keep them stone-free after initial management. Although this study is helpful in stratifying the workup for children with acute disease, it highlights the importance of initiating early treatment for these patients before long-term renal damage or complications occur.

A recent study performed by Cohen et al evaluated the healthcare costs of children with chronic medical complexity in the province of Ontario.^10^ They noted that despite these children accounting for <1% of the provincial pediatric population, one third of the pediatric healthcare expenditure is spent on them, with one factor attributed to the use of multiple medical specialties. With the MSC, specialty care may be consolidated and made cohesive both for patient convenience and improved patient understanding of their illness while decreasing the financial burden on the overall healthcare system.

Our study included a satisfaction survey in which both patients and parents agreed that the team approach provided consistent information when compared to separate specialty appointments. It also provided added efficiencies to families who would otherwise need to take more time off of work and pay for additional transportation if scheduling did not permit the same day appointments.

There are multiple limitations to our study. First, the patient population was limited and follow-up of the cohort was relatively short. Second, the clinic is highly specialized, and housed in a free-standing children’s hospital, and it has the necessary resources to manage these complex patients. These resources and specialists may not be available to other hospital systems looking to reproduce our work. Third, we understand that this study was performed in a single payer universal healthcare system, and costs will be significantly different compared to the US system. However, our work does show a decrease in the number of ED visits and surgeries after patients started attending the MSC, highlighting the importance of such a clinic in a system where costs are increasingly important. Additionally, one of the objectives of this study was to reduce imaging. However, although our data show decreased number of ED visits and surgeries, we did not see a significant decrease in the number of ultrasounds after the MSC clinic started. We believe this finding could be due to the relatively short follow-up period in the study. Longer-term data are needed to fully assess if the MSC contributes to a reduction in imaging. Fourth is the low response rate to the satisfaction surveys. The majority of surveys were collected at the clinic appointment to avoid any recall bias; however, for future work, a more immediate and useful delivery system would be to have participants complete the survey online and eliminate the need for return mail. Furthermore, in this study, we aimed to determine the impact of an MSC in children with a high stone burden. The rationale of including children followed for at least 2 years by individual clinics was to capture patients with a high burden stone disease. We acknowledge that this could represent potential selection bias. This limitation could have been addressed by comparing data of those who were directly referred to the multidisciplinary clinic to those who were previously followed in individual clinics following diagnosis; however, the sample size is small in our study population. Future investigations with larger cohorts will be required. Finally, we limited our analysis to 1 single surgical procedure, ureteroscopy, because that is the most common surgical procedure for stone removal in our institution. All patients with high stone burden who underwent surgery had at least one. Additionally, the number of other procedures such as extracorporeal shock wave lithotripsy and percutaneous nephrolithotomy was small, not allowing appropriate comparison. Physically combining specialties can be challenging when finding a common clinic space. Once accomplished, the benefits of the MSC for our patients surpassed the challenges. Longer-term data will help elucidate if the clinic has beneficial effects over a child’s lifetime. As the clinic continues, the number of surgeries and interventions will hopefully diminish.

## CONCLUSIONS

Children with metabolic stone disease benefit from an MSC with fewer ER visits and surgical interventions. Parents and families are satisfied with a more efficient model of care that provides consistent messaging about expected and needed care.

## DISCLOSURE

The authors have no financial interest to declare in relation to the content of this article.

## Supplementary Material


