# Supplementary material for: Cost Effectiveness and Impact in Quality of Care of a Pediatric Multidisciplinary Stone Clinic
Source: Pediatr Qual Saf. 2021 Sep 24;6(5):e474. doi: 10.1097/pq9.0000000000000474 (PMC8476049; doi:10.1097/pq9.0000000000000474)
Supplement: Supplementary file 1 [file pqs-6-e474-s001.pdf]

FIGURE LEGENDS

Supplemental Material

Figure 1. Survey of MSC handed out in person or sent to families for evaluation.

Date	________________________________
Who is filling out the form today?	◯ Parent<br>◯ Child
Child's age(years)	◯ 0-5<br>◯ 6-10<br>◯ 11-15<br>◯ 16-18
Does your child have an identified metabolic cause for his/her renal stone(s)?	◯ Yes<br>◯ No<br>◯ Unsure
If you answerend YES to above question, what is the name of the condition?	☐ Hypercalciuria (high urine calcium)<br>☐ Cystinuria<br>☐ Primary hyperoxaluria<br>☐ Hypocitraturia<br>☐ Hyperuricosuria (high urine uric acid)<br>☐ I don't remember<br>☐ Other
If you answerend OTHER to above question, what is the name of the condition?	________________________________
How long has your child been seen in the Combined Stone Clinic?	◯ First visit<br>◯ 3-6 months<br>◯ 6 months-1 year<br>◯ More than 1 year
Approximately how many visits has your child had to any Emergency Department (ED) due to renal stones :	◯ 0<br>◯ 1<br>◯ 2<br>◯ 3<br>◯ ≥4<br>◯ unsure
Approximately how many visits has your child had to any Emergency Department (ED) due to renal stones over the last year:	◯ 0<br>◯ 1<br>◯ 2<br>◯ 3<br>◯ ≥4<br>◯ unsure:
How many surgical procedures has your child had for stone removal so far?	◯ 0<br>◯ 1<br>◯ 2<br>◯ 3<br>◯ ≥4
How many surgical procedures has your child had for stone removal over the last year?	◯ 0<br>◯ 1<br>◯ 2<br>◯ 3<br>◯ ≥4
Does your child take any medication for renal stones?	◯ Yes<br>◯ No

What medication does your child take? Please mark all options that apply.

- ☐ Potassium citrate (K-citra, K-lyte, urocit K)
- ☐ Hydrochlorothyazide
- ☐ Thiola
- ☐ Captopril
- ☐ Vitamin B6 (pyridoxine)
- ☐ Allopurinol
- ☐ Phosphate
- ☐ Penicillamine
- ☐ Other:________________

If you answered other to above question, what is the name of the medication?

____

How long has your child been taking these medications?

- ◯ 0-6 months
- ◯ > 6 months - 1 year
- ◯ >1-2 years
- ◯ >2 years

How often did your child skip/miss doses of his/her medication over the last year?

- ◯ Never
- ◯ 1-2/month
- ◯ 1-2/week
- ◯ 3 or more times/week
- ◯ Uncertain

If uncertain, why? Please comment

____

What is your child's prescribed daily fluid intake?

- ◯ 1-1.5 liters
- ◯ greater than 1.5 to 2 liters
- ◯ greater than 2 to 2.5 liters
- ◯ More than 2.5 to 3liters
- ◯ greater than 3 liters
- ◯ Uncertain

If uncertain, why? Please comment

____

Is your child meeting his/her daily fluid intake goals?

- ◯ Yes
- ◯ No
- ◯ Uncertain

If uncertain, why? Please comment

____

How often did your child reach their daily recommended fluid intake?

- ◯ Never
- ◯ Rarely
- ◯ 1-2 times/week
- ◯ 3 or more times /week
- ◯ Every day

How often did your child eat fast food or frozen pre-packed food over the last year?

- ◯ Never
- ◯ 1-2 times/month
- ◯ 1-2 times/week
- ◯ 3 or more times/week
- ◯ Every day

Does your child need to follow diet restrictions other than salt restriction?

- ◯ Yes
- ◯ No

If you answered YES to above question, how often does he/she follow those diet restrictions?

- ◯ Never
- ◯ rarely
- ◯ 1-2times/week
- ◯ ≥3 times/week
- ◯ Every day
- ◯ Uncertain

If uncertain, why? Please comment

____

Has the information given today been consistent amongst all team members?

- ◯ Yes
- ◯ No
- ◯ Partially

Please Comment: ____________________ ______________________________ Have all your questions been answered during this visit?

- ◯ Yes
- ◯ No
- ◯ Partially

Please comment ______________________________

Please check all that apply to your experience in the Combined Stone clinic, seeing both Nephrology and Urology teams at the same visit:

- ☐ Time-saving (coming to hospital less times)
- ☐ Teams are integrated and give consistent information
- • It helps me better understand my child's condition
- ☐ All aspects of treatment are discussed at once
- ☐ Longer length of visit
- ☐ It is overwhelming when too much information is given at once
- ☐ Separate clinic visits with urology and nephrology would make my child's plan of care easier to understand
- ☐ None of the above

Please comment ______________________________

Do you have any other comments or suggestions about the Combined Urology-Nephrology Stone Clinic? ______________________________
